# Cathepsin B Is Required for NLRP3 Inflammasome Activation in Macrophages, Through NLRP3 Interaction

**DOI:** 10.3389/fcell.2020.00167

**Published:** 2020-03-31

**Authors:** Angélique Chevriaux, Thomas Pilot, Valentin Derangère, Harmonie Simonin, Pierre Martine, Fanny Chalmin, François Ghiringhelli, Cédric Rébé

**Affiliations:** ^1^INSERM Lipid Nutrition and Cancer UMR 1231, Dijon, France; ^2^Centre Georges François Leclerc, Dijon, France; ^3^Platform of Transfer in Cancer Biology, Centre Georges François Leclerc, Dijon, France; ^4^University of Bourgogne Franche-Comté, Faculty of Medicine, Dijon, France

**Keywords:** NLRP3, Cathepsin B, macrophages, IL-1β, caspase-1

## Abstract

The mechanisms leading to NOD-leucine rich repeat and pyrin containing protein 3 (NLRP3) inflammasome activation are still debated. It is well established that oligomerized NLRP3 interacts with apoptosis associated Speck-like protein containing a CARD domain (ASC) which polymerizes into filaments recruiting procaspase-1, leading to its activation. However, pathways triggering NLRP3 activation, such as potassium efflux, ROS production or lysosomal permeabilization, can be required or not, depending on the activators used. Here we proposed to evaluate the importance of Cathepsin B on NLRP3 inflammasome assembly and activation. Using Cathepsin B^–/–^ BMDMs (Bone Marrow-Derived Macrophages), we first show that Cathepsin B is required for caspase-1 activation, IL-1β production and ASC speck formation, upon treatment with different types of NLRP3 activators, i.e., ATP, nigericin or crystals. Moreover, in these conditions, Cathepsin B interacts with NLRP3 at the endoplasmic reticulum (ER) level. To conclude, different NLRP3 activators lead to Cathepsin B interaction with NLRP3 at the ER level and to subsequent caspase-1 activation.

## Introduction

Inflammasomes are intracellular complexes constituted of a receptor and an adaptor that enable the recruitment and activation of pro-inflammatory caspases such as caspase-1 and the maturation of pro-inflammatory cytokines such as IL-1β or IL-18 (Guo et al., 2015). The receptors, called NOD-like receptors are activated by a wide diversity of stimuli called pathogen-associated molecular patterns (PAMPs) or danger-associated molecular patterns (DAMPs) or environmental stresses. NLR family is characterized by specific domains. The central NACHT domain is responsible for ATP-dependent oligomerization, whereas the C-terminal leucine reach repeat (LRR) domain has a role in ligand detection and complex autoregulation. On the N-terminal, these receptors contain a CAspase recruitment domain (CARD) or a PYrin Domain (PYD) implicated in protein/protein interactions involved in signal transduction (Davis et al., 2011). Activated receptors can recruit either pro-caspases or adaptors proteins (via the PYD) that will in turn recruit pro-caspases. The phylogenic analysis of the NACHT domain distinguishes three sub-families of NLRs: the NOD, the NLRP or IPAF (Davis et al., 2011).

The NOD-leucine rich repeat and pyrin containing protein 3 (NLRP3) inflammasome is the most described complex. It is constituted by NLRP3, the adaptor apoptosis associated Speck-like protein containing a CARD domain (ASC) and pro-caspase-1. In absence of any stimuli, NLRP3 is maintained in an inactive form at the endoplasmic reticulum (ER) level. ASC is mainly localized in mitochondria. In the presence of activators, the intracellular concentration of NAD^+^ decreases, leading to sirtuin 2 (SIRT2) inactivation and accumulation of acetylated α-tubulin responsible for proximity between mitochondria and ER (Misawa et al., 2013). This enables ASC interaction with NLRP3, through the PYD and ASC polymerization into filaments (Lu et al., 2014). This oligomerized complex can recruit pro-caspase-1 via the CARDs of ASC and pro-caspase-1, leading to the cleavage and activation of pro-caspase-1. The active caspase-1 will in turn cleave pro-IL-1β and pro-IL-18 to produce mature IL-1β and IL-18.

The process leading to NLRP3 inflammasome activation consists in two different steps: priming and activation. The first step begins with recognition by pattern recognition receptors (PRRs) of extracellular molecules such as lipopolysaccharides (LPS), tumor necrosis factor α (TNFα), or IL-1β (Schroder et al., 2012). This will have two main consequences: NF-κB activation, allowing NLRP3 and IL-1β transcription (Bauernfeind et al., 2009) and deubiquitination of NLRP3 LRR domain, required for its activation (Juliana et al., 2012; Py et al., 2013). The activation step is engaged when cells are exposed to endogenous or exogenous molecules. This includes ATP or the bacterial toxin nigericin, that both induce a decrease in intracellular potassium concentrations or crystal structures phagocytosis, such as MonoSodium Urate (MSU) (Mariathasan et al., 2006; Martinon et al., 2006; Petrilli et al., 2007). Once constituted, the NLRP3 inflammasome is then secreted in cell supernantant and can amplify the inflammatory response by activating inflammasomes and caspase-1 in neighboring cells (Baroja-Mazo et al., 2014; Franklin et al., 2014).

Some steps of NLRP3 inflammasome activation pathway still remain unclear, such as the importance of Cathepsin B (Campden and Zhang, 2019). We have recently shown, that Cathepsin B interaction with NLRP3 was required for 5-FU (5-FluoroUracil) or gemcitabine-mediated caspase-1 activation in myeloid-derived suppressor cells (MDSCs) (Bruchard et al., 2013). To determine whether this observation could be generalized, we have analyzed if Cathepsin B could interact with NLRP3 in macrophages exposed to classical sterile activators. In this study, we showed that Cathepsin B was required for caspase-1 activation induced by many different NLRP3 inflammasome activators. Moreover, Cathepsin B interacts with NLRP3 at the ER level.

## Materials and Methods

### Reagents

LPS (L3024), ATP (A7699) and nigericin (N7143) were purchased from Sigma-Aldrich. SiO_2_ (tlrl-sio), MSU (tlrl-msu) and CPPD (tlrl-cppd) were purchased from InvivoGen.

### Cell Culture

Human myeloid THP-1 cells were obtained from the American Type Culture Collection (ATCC) and were grown in RPMI 1640 with ultraglutamine (Lonza) supplemented with 10% (vol/vol) fetal bovine serum (FBS; Lonza) and with Pen/Strep Amphotericin B (PSA, Lonza) 1%, in an atmosphere of 95% air and 5% CO_2_ at 37°C. Cells were primed with 300 ng/mL of LPS (Sigma-Aldrich) for 3 h and treated with ATP (5 mM) for 30 min.

### Mice

All animals were bred and maintained according to both the FELASA and the Animal Experimental Ethics Committee Guidelines (University of Burgundy, France). Animals used were between 6 and 22 weeks of age. Female C57BL/6 mice (aged 6 to 8 weeks) were obtained from Charles River Laboratories and C57BL/6 Cathepsin B^–/–^ mice from T. Reinheckel, bred and maintained in the “Cryopréservation, Distribution, Typage et Archivage Animal (CDTA-Orléans, France).”

### Mouse Bone Marrow-Derived Macrophages (BMDMs)

C57BL/6 mice bone marrow cells were isolated from tibias and femurs as previously described (Martine et al., 2019) and cultured for 6 days on plastic plates in RPMI 1640 medium with ultraglutamine (Lonza) supplemented with 10% (vol/vol) fetal bovine serum (FBS; Lonza) in the presence of 50 ng/mL of M-CSF (216-MC – R&D systems), in an atmosphere of 95% air and 5% CO_2_ at 37°C. Subsequently, floating cells were removed and macrophage differentiation was observed by fibroblast-like shape changes visualized with a Zeiss PrimoVert microscope. Differentiated cells were then primed with 300 ng/mL of LPS (Sigma-Aldrich) for 3 h and treated by different inflammasome activators: nigericin (30 min – 5, 10, 20, 40, 50, and 100 μM), ATP (30 min – 0.5, 1, 2, 5, 7, and 10 mM), SiO_2_, CPPD or MSU (6 h – 10, 20, 50, 100, 200, and 500 μg/mL).

### IL-1β Detection

Murine IL-1β was detected using the Mouse IL-1 beta/IL-1F2 DuoSet ELISA (DY401-05) kit from R&D Systems, as previously described (Martine et al., 2019) and according to manufacturer’s instructions. Briefly, 96-well plates were coated overnight at room temperature with 100 μL of diluted IL-1β capture antibody at 4 μg/mL. After washing three times, wells were blocked for 1 h. Then, 100 μL of samples or standards were incubated for 2 h at room temperature. After additional three washes, 100 μL of diluted detection antibody at 500 ng/mL was added at room temperature for 2 h. Detection was performed using streptavidin-coupled HRP and its substrate with a microplate reader set at 450 nm. Concentration was evaluated using a standard curve.

### Supernatant Precipitation

Cells (1,5 × 10^6^/500 μL) previously primed with LPS or not, were treated in OptiMEM without FBS. The supernatants were harvested by centrifugation at 400*g* for 5 min and precipitated. Methanol (500 μL) and chloroform (150 μL) were added and the samples were vortexed for 10 s. After centrifugation at 12 000g for 10 min, the aqueous phase (at the top) was discarded and 800 μL of methanol were added. Samples were vortexed and centrifuged at 12,000 *g* for 10 min and supernatants were removed. Pellets (containing proteins) were dried for 10 min at 37°C, mixed with 40 μL of loading buffer (125 mM Tris–HCl [pH 6.8], 10% β-mercaptoethanol, 4.6% SDS, 20% glycerol, and 0.003% bromophenol blue) and incubated at 95°C for 5 min.

### Immunoprecipitation

Immunoprecipitations were performed as previously described (Rebe et al., 2007). Cells (50 × 10^6^) were lysed in 1 mL lysis buffer (20 mM Tris [pH 7.5], 150 mM NaCl, 1% NP-40, 10% glycerol and complete protease inhibitor mixture [CPIM]) for 30 min on ice. After centrifugation at 14,000 *g* at 4°C for 30 min, supernatants were pre-cleared during 2 h at 4°C in the presence of 30 μL of mixed Sepharose 6B (6B100, Sigma Aldrich) and protein G (17-0618-01, Amersham). After centrifugation at 1000 *g* for 3 min the supernatant was incubated with anti-Cathepsin B (sc-6493, Santa Cruz) or anti-NLRP3 antibodies (AG-20B-0014, Adipogen) (2 μg/mL) at 4°C for 20 h and during the last hour with 40 μL of mixed Sepharose. The precipitates were washed 4 times in lysis buffer and analyzed by immunoblotting.

### Western Blotting

Whole-cell lysates were prepared as described previously (Courtaut et al., 2015), by lysing the cells in boiling buffer [1% SDS, 1 mM sodium vanadate, 10 mM Tris (pH 7.4)] in the presence of complete protease inhibitor mixture. Samples viscosity was reduced by sonication.

Whole-cell lysates or immunoprecipitation samples were separated by sodium dodecyl sulfate–polyacrylamide gel electrophoresis (SDS-PAGE), and electroblotted to a nitrocellulose membrane (GE Healthcare) in a borate buffer. After incubation for 2 h at RT by 5% non-fat milk in Phosphate-buffered saline (PBS)-0.1% Tween-20, membranes were incubated overnight with primary antibody diluted in PBS-milk-Tween. Membranes were then washed, incubated with secondary antibody for 30 min at RT and washed again before analysis with a chemiluminescence detection kit (Santa Cruz Biotechnologies).

Precipitated supernatants were also separated by SDS-PAGE and electroblotted to a nitrocellulose membrane in an ethanol-containing buffer. After incubation for 2 h at RT in 5% non-fat milk in PBS-0.5% Tween-20, membranes were incubated overnight with primary antibody diluted in PBS-milk-Tween, washed, incubated with secondary antibody for 2 h at RT, and washed again before analysis with a chemiluminescence detection kit (Amersham).

The following mouse mAbs were used: anti–β-actin (A1978) from Sigma-Aldrich, anti-NLRP3 (AG-20B-0014) and anti-caspase-1 (AG-20B-0044) from Adipogen. Rat pAbs anti-IL-1β (MAB-4011) from R&D Systems, rabbit pAbs anti-ASC (AG-25B-0006, Adipogen) from Enzo life sciences, anti-Cathepsin B and anti-GSDMD (Ab209845 – Abcam) as well as secondary Abs HRP-conjugated immunoglobulins (Jackson ImmunoResearch) were also used. Uncut western blots were shown in Supplementary Material.

### Flow Cytometry

Mouse bone marrow-derived macrophages were treated with indicated activators and incubated during the last 30 min with 75 nM of Lysotracker^TM^ Deep Red (Molecular Probes). Cells were then recovered and analyzed with a LSRII (BD Biosciences) and FlowJo software.

### Immunofluorescence (IF) and *in situ* Proximity Ligation Assay (PLA)

Experiments were performed as previously described (Derangere et al., 2014). Cells were treated with LPS and subsequently with different inflammasome activators. Cells were washed in PBS, fixed with 4% PFA at 4°C for 10 min and permeabilized using a PBS, 0.5% BSA, 0.1% Saponin (47036, Sigma Aldrich) buffer for 20 min at RT. Samples were incubated 2 h at RT with primary antibodies or with Ig as a control.

For IF experiments, cells were washed two times, and incubated with secondary Alexa568 conjugated anti-rabbit for 30 min at RT. For PLA experiments (Sigma-Aldrich DUO92007), after washing primary antibodies, cells were then incubated with the appropriate probes (Sigma Aldrich DUO92004 and DUO92002) during 1 h at 37°C and washed two times. Probes were then ligated for 30 min at 37°C, washed two times in Buffer A and amplified using the manufacturer’s polymerase for 100 min at 37°C in the dark.

For both experiments, cover glasses were mounted on a drop of Mounting Medium containing Dapi (Duo82040, Sigma Aldrich) for 15 min in the dark, on a microscopy slide (045796, Dutscher, Brumath, France). Slides were imaged using a CDD equipped upright microscope (Zeiss) and 63×, 1.4NA objective. Image analysis was performed using ImageJ software.

The following antibodies were used for IF and PLA: mouse anti-NLRP3 (AG-20B-0014, Adipogen), anti-caspase-1 (AG-20B-0044-C100, Adipogen), rabbit anti-ASC (AG-25B-0006, Adipogen), anti-Tom20 (sc-11415, Santa Cruz), anti-calreticulin (#12238, Cell Signaling), anti-Lamp-1 (sc-5570, Santa Cruz), goat anti-Cathepsin B (sc-6493, Santa Cruz), anti-mouse Alexa488 (A11029, Invitrogen), anti-rabbit Alexa568 (A11036, Invitrogen), and donkey anti-mouse Alexa568 (A10037, Invitrogen).

### Lactate Dehydrogenase (LDH) Release

Cell death was evaluated by measuring LDH release in the supernatant, using the CytoTox^®^ 96 Non-Radioactive Cytotoxicity Assay (Promega) according to manufacturer’s instructions. Briefly, after treatments, cell supernatants were transferred in a 96-well plate and were incubated with CytoTox^®^ 96 Reagent for 30 min at room temperature. Stop solution was then added and OD was measured at 490 nm. A maximum LDH release control was done by adding lysis solution on cells. Percentages of LDH release was calculated with the formula:% LDH release = (OD sample × 100)/OD maximum LDH release sample.

### Statistical Analysis

Results are shown as mean ± standard deviation (s.d.). Dataset comparisons were performed with GraphPad Prism 8, using paired Student’s *t* tests (test group compared to control group). All *P* values were two tailed.

## Results

### Cathepsin B Is Required for NLRP3 Inflammasome Activation

**FIGURE 1:**
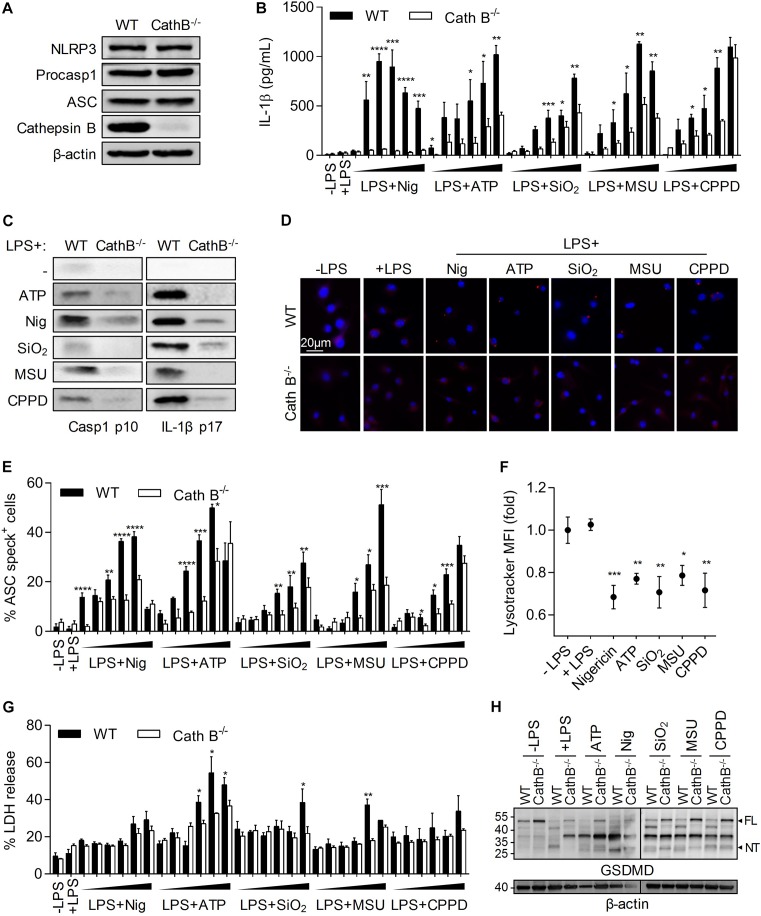
Cathepin B is required for NLRP3 inflammasome activation and IL-1β production. BMDMs were primed with 300ng/mL of LPS for 3 h. Cells were then left untreated **(A)**, or treated by different inflammasome activators: nigericin (30 min – 5, 10, 20, 40, 50, and 100 μM), ATP (30 min – 0.5, 1, 2, 5, 7, and 10 mM), SiO_2_, CPPD, and MSU (6 h – 10, 20, 50, 100, 200, and 500 μg/mL). Unprimed cells (-LPS) were also used **(B,E,G)**. In other experiments cells were treated with nigericin (30 min – 40 μM), ATP (30 min – 5 mM), SiO_2_, CPPD and MSU (6 h – 100 μg/mL) **(C,D,F,H)**. **(A)** Expression of indicated proteins was evaluated in BMDMs lysates from WT or Cathepsin B^–/–^ C57BL/6 mice. **(B)** IL-1β content in supernatant was measured by ELISA. **(C)** Cleavage of caspase-1 and IL-1β was evaluated in supernatants by western blot. **(D,E)** ASC specks formation was determined by immunofluoresence. **(F)** Lysosome destabilization was evaluated by LysoTracker staining and flow cytometry analysis. **(G)** Cell death was monitored by measuring LDH release in the supernatant. **(H)** Gasdermin D (GSDMD) cleavage was evaluated in cell lysates by western blot. Numbers indicate MW in kDa. β-actin was used as loading control. FL, full length; NT, N-ter cleavage fragment. Data represent either one representative experiment or the mean of at least three independent experiments ± SD. Statistics compared WT and Cathepsin B^–/–^ cells with similar treatments **(B,E,G)** or untreated cells with treated cells **(F)**. *p < 0.05, **p < 0.01, ***p < 0.005, ****p < 0.001, no symbol, not significant.

To determine the importance of Cathepsin B in NLRP3 inflammasome activation, we used, BMDMs from Cathepsin B KO or WT mice. While the expression of Cathepsin B was totally disrupted in BMDMs from KO mice, the expression of the main NLRP3 inflammasome components was unchanged when compared to WT BMDMs (Figure 1A). Moreover, Cathepsin B absence didn’t affect macrophage differentiation (data not shown). Cells were primed with LPS and treated *in vitro* with different NLRP3 inflammasome activators, i.e., nigericin (Nig), ATP, silica (SiO_2_), MonoSodium Urate (MSU) and calcium pyrophosphate dihydrate crystals (CPPD). The IL-1β production induced by increasing concentrations of different activators in WT BMDMs was inhibited in Cathepsin B KO cells (Figure 1B). Caspase-1 activation and IL-1β production in Cathepsin B KO BMDMs supernatants after NLRP3 inflammasome activators was also partially or totally inhibited (Figure 1C). Finally, the lack of Cathepsin B also inhibited the NLRP3 inflammasome complex formation as shown by the decrease of ASC oligomers in Cathepsin B KO BMDMs treated with different concentrations of activators tested (Figures 1D,E). Then, the importance of the lysosome/Cathepsin B pathway was confirmed by showing the lysosomal destabilization after different activators treatments (Figure 1F). Finally, since NLRP3 inflammasome and caspase-1 activation can lead to pyroptotic cell death (Derangere et al., 2014; He et al., 2015; Shi et al., 2015), we compared the impact of activators tested on pyroptosis features between WT and Cathepsin B KO BMDMs. We showed that the capacity of WT cells to release LDH in the supernatant under increasing concentrations of inflammasome activators was partially decreased in Cathepsin B KO cells, especially for higher doses of ATP, SiO_2_ or MSU (Figure 1G). Moreover a cleavage of Gasdermin D (GSDMD) appeared under some conditions in WT BMDMs and is less important in Cathepsin B KO cells (Figure 1H). Altogether, these results demonstrate the importance of Cathepsin B in NLRP3 inflammasome-triggered ASC oligomerization, caspase-1 activation and IL-1β maturation.

### Cathespin B Interacts With NLRP3 Upon NLRP3-Inflammasome Activators Treatment

**FIGURE 2:**
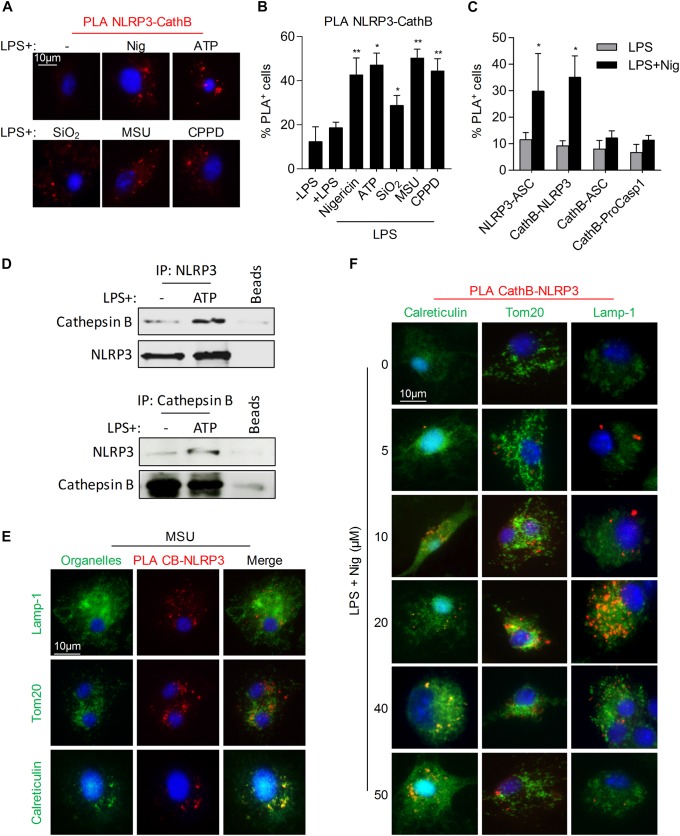
Cathepsin B and NLRP3 interact in monocytes/macrophages under inflammasome activation. **(A,B)** BMDMs were primed with 300 ng/mL of LPS for 3 h. Cells were then treated with nigericin (30 min – 40 μM), ATP (30 min – 5 mM), SiO_2_, CPPD or MSU (6 h – 100 μg/mL) and stained with anti-NLRP3 and anti-Cathepsin B for PLA experiments. Representative images **(A)** and percentages of cells with fluorescent dots were evaluated **(B)**. **(C)** Primed BMDMs were treated with nigericin (30 min – 40 μM), stained with indicated antibodies and assayed for PLA. **(D)** LPS primed THP-1 cells treated or not with ATP (30 min – 5 mM) were lysed, immunoprecipitated with anti-NLRP3 or anti-Cathepsin B antibodies and analyzed by western blot. **(E,F)** BMDMs treated as indicated were stained either with anti-calreticulin, anti-Tom20 or anti-Lamp-1 and specific secondary antibodies (green), with DAPI (blue) and with anti-NLRP3 and anti-Cathepsin B antibodies and assayed for PLA (red). Representative images are shown. Data represent either one representative experiment or the mean of at least three independent experiments ± SD. Statistics compared untreated cells with treated cells. *p < 0.05, **p < 0.01, no symbol, not significant.

We previously demonstrated that Cathepsin B/NLRP3 interaction was required for 5-FU and gemcitabine-induced caspase-1 activation in MDSCs (Bruchard et al., 2013). Using PLA experiments, we showed a proximity of Cathepsin B with NLRP3 in murine BMDMs exposed either to nigericin, ATP, SiO_2_, MSU, or CPPD (Figures 2A,B). Moreover, the interaction of Cathepsin B seemed to occur only with NLRP3, as we failed to observe any association with ASC or pro-caspase-1 (Figure 2C). The interaction between Cathepsin B and NLRP3 was confirmed by immunoprecipitations in THP-1 cells treated with ATP (Figure 2D). NLRP3 inflammasome assembly was shown to occur upon proximity of mitochondria to ER, allowing ASC and NLRP3 interaction (Misawa et al., 2013). To determine the localization of Cathepsin B/NLRP3 interaction, we performed PLA experiments associated to a co-staining of Lamp-1 (lysosomes), Tom20 (mitochondria) or calreticulin (ER) (Figures 2E,F). As observed, there was no co-localization of PLA red dots neither with Lamp-1 nor with Tom20, in BMDMs treated with MSU (Figure 2E) or with increasing concentrations of nigericin (Figure 2F), suggesting that NLRP3/Cathepsin B interaction did not occur in lysosomes nor in mitochondria. However, a co-localization of PLA dots with calreticulin was observed, showing that NLRP3/Cathepsin B interaction occurred at the ER level. Altogether, these results suggest that cathepsin B interacts with NLRP3 at the ER level in cells treated with NLRP3 inflammasome activators.

## Discussion

Literature remains elusive concerning the importance of Cathepsin B in NLRP3 inflammasome activation in macrophages (Campden and Zhang, 2019). Cathepsin B is cleaved/activated along monocyte differentiation into macrophages and participates with caspases to the acquisition of a mature phenotype (Rebe et al., 2007; Guery et al., 2011). However, we and others have shown that Cathepsin B remains in the lysosomes and is released into the cytoplasm upon exposure to some NLRP3 inflammasome activators (Jin and Flavell, 2010; Bruchard et al., 2013). Cathepsin B is only catalytically active at acidic pH (Turk et al., 2012), therefore its role in the cytoplasm might be independent of its enzymatic activity, excluding a possible proteolysis of inflammasome components and/or regulators. This is supported by the absence of Cathepsin B targets degradation (such as Bcl-2, Bcl-XL and Mcl-1) in macrophages treated by nigericin or ATP (data not shown) and by the fact that LPS can dampen Cathepsin B activity (Guery et al., 2011). In this context, many studies proposed opposing conclusions about the importance of Cathepsin B in NLRP3 inflammasome activation, using the inhibitor CA-074Me [(L-3-*trans*-(Propylcarbamoyl)oxirane-2-carbonyl)-L-isoleucyl-L-proline Methyl Ester] (Hentze et al., 2003; Hornung et al., 2008; Rajamaki et al., 2010; Riteau et al., 2012; Orlowski et al., 2015). It can be explained by the lack of specificity. First, it may inhibit Cathepsin B in the lysosome arguing for a role of this enzyme in this organelle. Here we provide evidence for the importance of Cathepsin B in the cytoplasm, i.e., to interact with NLRP3. Moreover, CA-074Me can also inhibit other cathepsins, such as Cathepsin L (Montaser et al., 2002). A compensatory mechanism was previously proposed between cathepsins B, C, S and Z (Orlowski et al., 2015). Moreover, cathepsins L, Z, and S were shown to inhibit IL-1β production by macrophages under cholesterol crystals, ATP, MSU, or Alum treatments (Duewell et al., 2010; Oleszycka et al., 2016; Allan et al., 2017). However, these studies didn’t explore whether these enzymes are required for NLRP3 inflammasome activation and/or IL-1β secretion. Here we show that cathepsin B deficiency inhibits ASC oligomerization, caspase-1 activation and IL-1β production. Moreover, CA-074Me was also described to inhibit MSU-induced IL-1α and IL-6 production (Gicquel et al., 2015). To show the importance of Cathepsin B, other studies have used Cathepsin B deficient mice. In some cases, the lack of Cathepsin B seemed to have only mild effects on caspase-1 activation and IL-1β maturation-induced by MSU or alum, while in other cases it seemed to be required (Dostert et al., 2009; Riteau et al., 2012; Hari et al., 2014; Orlowski et al., 2015). In our experiments, macrophages from Cathepsin B-deficient mice were unable to release active caspase-1 and mature IL-1β under several treatments, i.e., ATP, nigericin, SiO_2_, MSU or CPPD. These discrepancies may be explained by experimental differences such as (1) the type of myeloid cells (peritoneal macrophages or BMDMs), (2) the LPS priming (origin and concentration), and (3) the NLRP3 activators (concentrations and treatment duration).

We previously showed with recombinant proteins, that Cathepsin B interacts directly with the LRR domain of NLRP3 (Bruchard et al., 2013). However, the capacity of Cathepsin B to interact with NLRP3 in macrophages had not been evaluated yet. Here, we used several NLRP3 inflammasome activators with different signaling pathways. Some of them such as SiO_2_, MSU, or CPPD must be phagocytized to mediate lysosome destabilization, like 5-FU and gemcitabine in MDSCs (Leemans et al., 2011; Bruchard et al., 2013). The link between these activators and Cathepsin B requirement is quite consistent. However, the involvement of Cathepsin B in other activator-mediated NLRP3 inflammasome activation, such as nigericin (Streptomyces-derived toxin) or ATP (purinergic receptor ligand) is less evident. Here we highlighted Cathepsin B as a key component of the signaling pathway for different NLRP3 activators, which target macrophages at different levels.

Thus the classical schemes describing the activation of NLRP3 through either potassium efflux, ROS production or lysosomal permeabilization must be re-evaluated. Actually, the purinergic pathway has already been described to induce ROS production and the lysosmal/Cathepsin B pathway can activate the purinergic and the ROS pathways (Hornung et al., 2008; Zhou et al., 2010; Riteau et al., 2012). The lysosome destabilization was shown to enable pannexin 1 opening and ATP release in the extra-cellular space. Then, ATP will activate NLRP3 through fixation to its receptor P2RX7 and potassium efflux (Riteau et al., 2012). On the other hand, oxidative stress can be induced by Cathepsin B and will enable TXNIP [Thioredoxin (TRX)-Interacting Protein] to interact with NLRP3 to activate it (Zhou et al., 2010). However, the exact place of the NLRP3/Cathepsin B complex in these pathways need further experiments to be clarified.

Another major point in NLRP3 inflammasome formation was the subcellular localization of the different partners and their merge along activation. Different studies agreed on a cytoplasmic/ER localization for NLRP3 and a cytoplasmic/mitochondrial localization for ASC in untreated cells. Under stimulation, the interaction of NLRP3 with ASC occurs at the mitochondria/ER level (Zhou et al., 2011; Misawa et al., 2013; Subramanian et al., 2013). This interaction is allowed by tubulin acetylation and organelle moving (Misawa et al., 2013). To better characterize NLRP3 inflammasome formation, we showed here that Cathepsin B/NLRP3 interaction happens at ER level and not at the lysosomal or mitochondrial level. We conclude that Cathepsin B should be localized out of the lysosome to reach NLRP3 at its inactivated localization site, ER, and that Cathepsin B/NLRP3 never relocalize to the mitochondria. Moreover, Cathepsin B seems not to interact with ASC nor pro-caspase-1. All these results might suggest that the Cathepsin B/NLRP3 interaction is transient and that Cathepsin B is not a former member of the NLRP3 inflammasome complex.

Altogether our study brings a new mechanistic detail in the NLRP3 inflammasome activation pathway by several activators. Moreover, it raises the importance of Cathepsin B and the interest to develop inhibitor peptides targeting its interaction with NLRP3, an early and common event, to inhibit IL-1β production in inflammatory diseases or cancer.

## Data Availability Statement

All datasets generated for this study are included in the article/Supplementary Material.

## Ethics Statement

The animal study was reviewed and approved by the Animal Experimental Ethics Committee Guidelines (University of Burgundy, France).

## Author Contributions

AC, TP, HS, VD, PM, and FC performed the experiments. CR designed the study and wrote the manuscript. AC, TP, and FG corrected the manuscript. All authors contributed to manuscript revision, read, and approved the submitted version.

## Conflict of Interest

The authors declare that the research was conducted in the absence of any commercial or financial relationships that could be construed as a potential conflict of interest.
