# Supplementary figures and images for: Cathepsin B Is Required for NLRP3 Inflammasome Activation in Macrophages, Through NLRP3 Interaction
Source: Front Cell Dev Biol. 2020 Mar 31;8:167. doi: 10.3389/fcell.2020.00167 (PMC7162607; doi:10.3389/fcell.2020.00167)

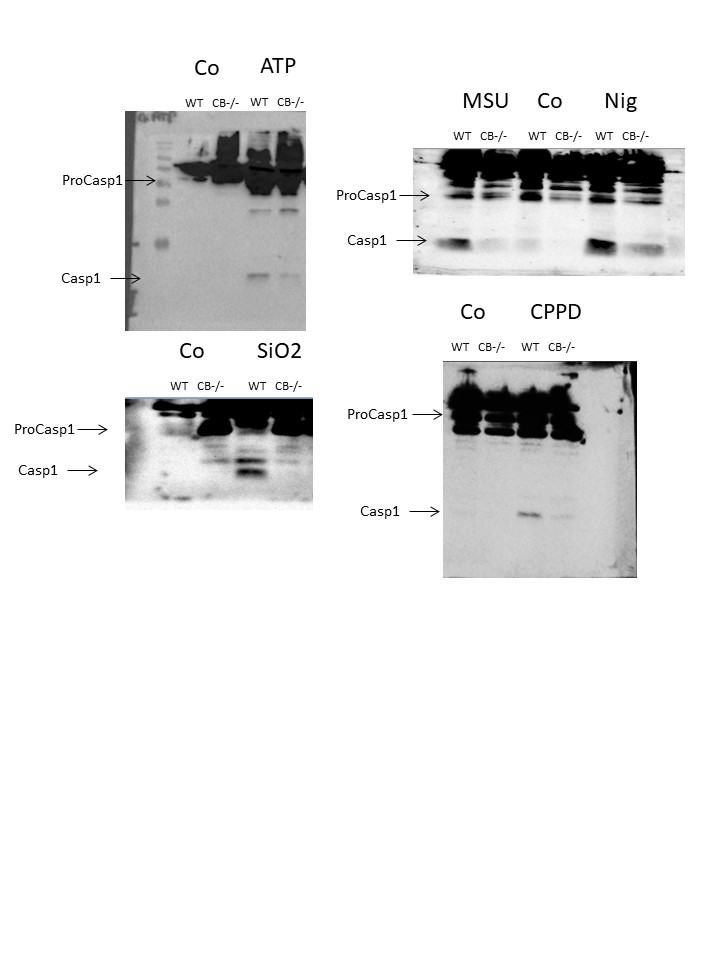

Supplement: Supplementary file 1 [file Image_1.jpeg]

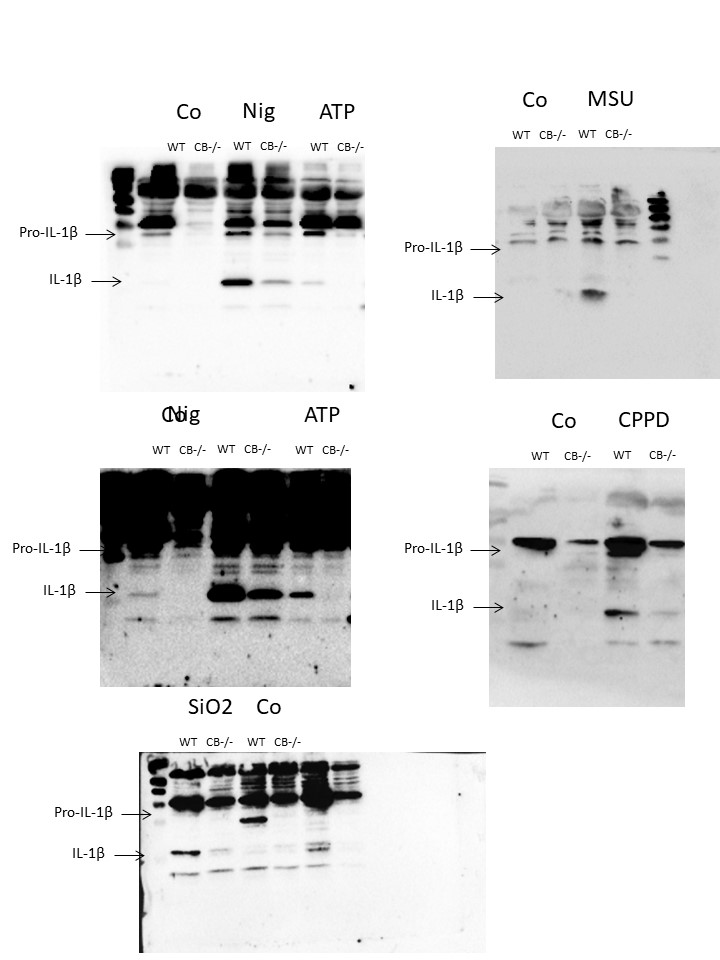

Supplement: Supplementary file 2 [file Image_2.jpeg]

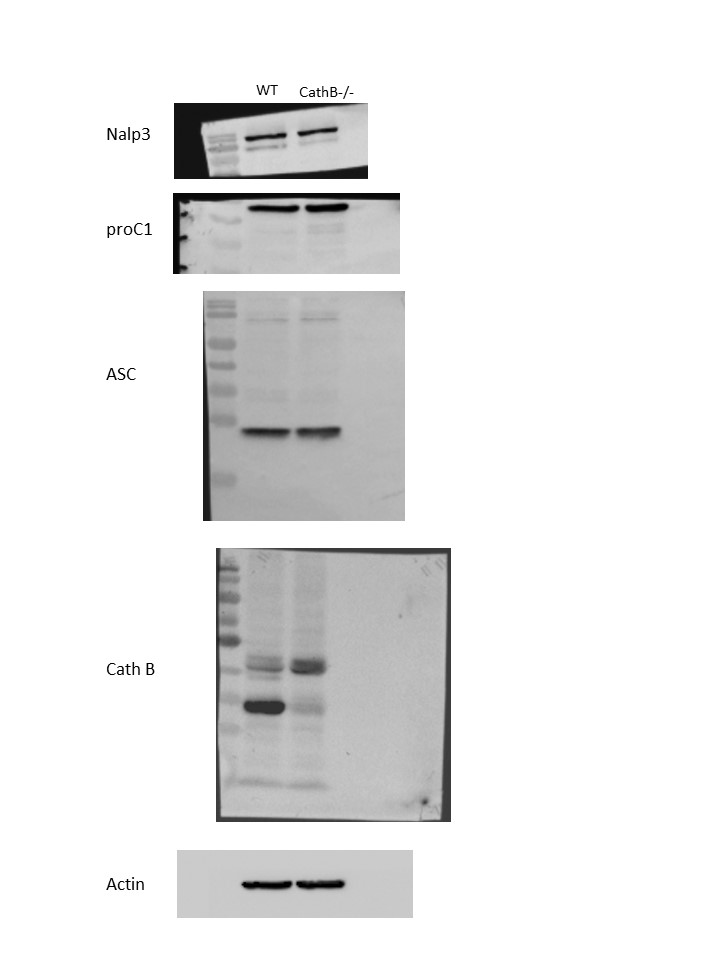

Supplement: Supplementary file 3 [file Image_3.jpeg]

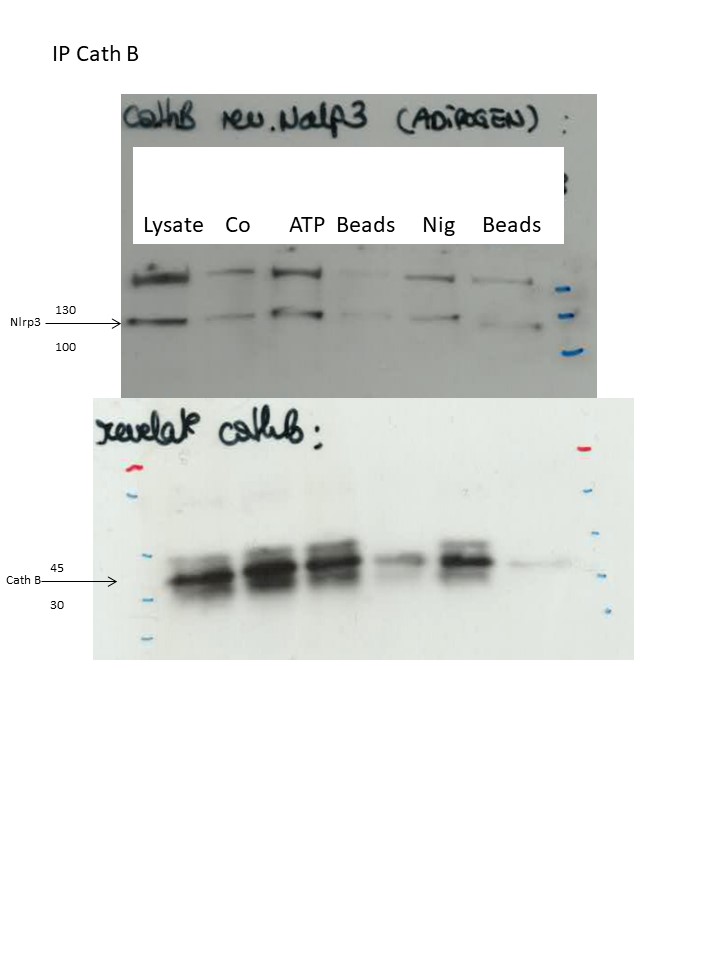

Supplement: Supplementary file 4 [file Image_4.jpeg]

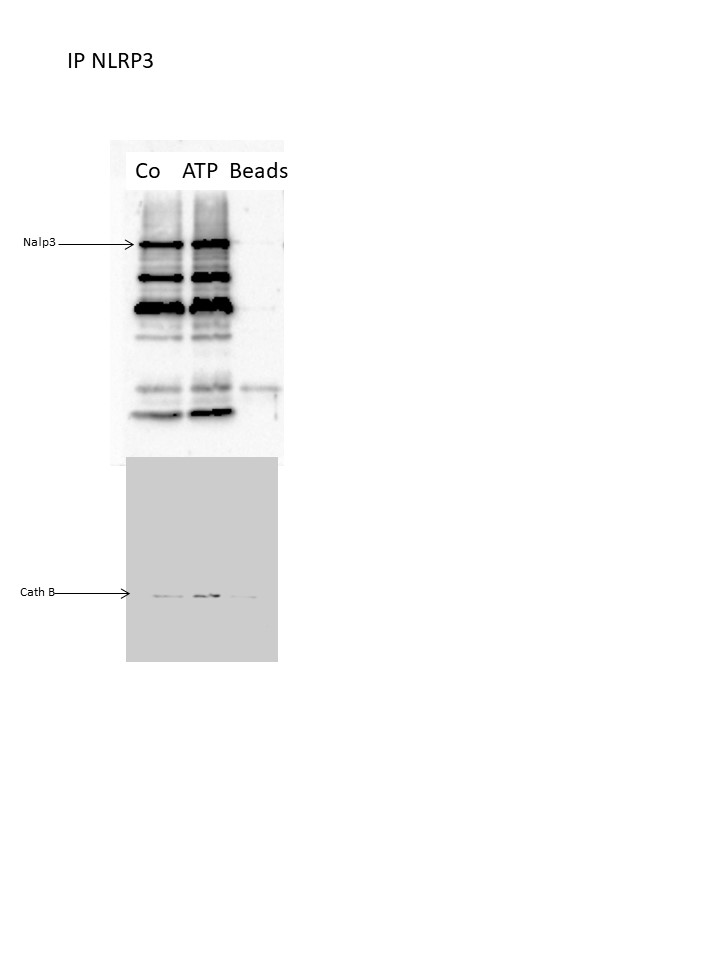

Supplement: Supplementary file 5 [file Image_5.jpeg]
